# Supplementary material for: Smallpox outbreak scenarios and reactive intervention protocols: A mathematical model-based analysis applied to the Republic of Korea
Source: PLoS One. 2026 Feb 10;21(2):e0315072. doi: 10.1371/journal.pone.0315072 (PMC12890121; doi:10.1371/journal.pone.0315072)
Supplement: Attachment — Submitted filename: CommentsToAuthor_PONE-D-24-53297.pdf [file pone.0315072.s001.pdf]

Comments to the Authors

Manuscript ID: PONE-D-24-53297

I have reviewed this manuscript from the perspective of a mathematical epidemiologist who has experience in infectious disease modelling, though with no prior experience in modelling the transmission dynamics of smallpox.

This study created a mathematical model to analyse potential smallpox epidemics, considering factors like age, contact patterns and intervention strategies (contact tracing, ring vaccination and mass vaccination). Carrying out model simulations applied to the setting of the Republic of Korea, the scenarios that underwent consideration suggest current isolation bed capacity would likely be sufficient, but extreme scenarios could overwhelm it. The study highlights the importance of non-pharmaceutical interventions and strategic vaccination prioritisation for outbreak control, offering guidance for public health officials preparing for bioterrorism and other infectious disease outbreaks.

In my opinion, the manuscript would benefit from edits in the following areas.

Open code: There was no link given to a code repository associated with the study. To enable replication by others, I would strongly encourage that a publicly accessible code repository is made containing the model simulation scripts and scripts to reproduce the figures presented in the manuscript.

Title: I suggest that the title is amended to reflect that the modelling work is applied in the context of the Republic of Korea. E.g. Smallpox bioterrorism scenarios and reactive intervention protocol: mathematical model-based analysis applied to the Republic of Korea.

Introduction

The current L344-345 in the discussion – “By 2024, the ROK plans to expand the number of negative-pressure isolation beds to 3500 to respond to emerging infectious diseases” – I would deem as relevant contextual information to provide the reader within the Introduction.

<u>References to previous modelling studies</u>: These were all from before 2010. There appear to be more recent modelling studies that the authors may consider adding to the paragraph on previous modelling studies (L81-91):

- B Mohanty *et al.* (2020). Modelling the impact of a smallpox attack in India and influence of disease control measures. *BMJ open*, **10**(12), e038480. https://doi.org/10.1136/bmjopen-2020-038480
- V. Costantino, M. Kunasekaran & C.R. MacIntyre. (2021). Modelling of optimal vaccination strategies in response to a bioterrorism associated smallpox outbreak. *Human Vaccines & Immunotherapeutics*, **17**(3), 738-746. https://doi.org/10.1080/21645515.2020.1800324

Methods

Contact structure: The reader is currently directed to a supporting reference for the age-group contact data. Given the assumptions being made between the close contacts and social contacts, I personally would find it very helpful to have a two-panel figure visualising the age-group contact structure, with one panel showing the values used for close contacts and the other panel the values used for social contacts.

Sensitivity analysis: For reproducibility purposes, a description of the sensitivity analysis conducted should be added to the Methods (including moving what is currently expressed on L312-321 from the Results to the Methods). I recommend the range of values considered for the parameter sensitivity analysis to also be added to Table 1, along with citing additional references as necessary to support the stated parameter value ranges.

Programming language and computing resources information: What was the programming language, and its version, used to perform the model simulations? Were there any additional packages that were used? Please add this information as part of the methods. Please also state if any high-performance computing resource was used for this modelling study.

L110-111: The text states: "we categorized the population into 16 age groups, each spanning 5 years, ranging from 0–4 years to 65 years and older in the Republic of Korea". However, that age range would only give 14 age groups. Table 2 later shows 5-year age groups up to 75 years and older. Please revise the text to correspond with the information in Table 2.

Table 2: I suggest this is turned into a figure. For example, a horizontal bar plot with a bar for each age group. The value can then still be stated on the figure, potentially within the bar itself or to the right-hand side of the bar.

Results

Subheadings: The baseline scenario results section contains multiple figures and multiple pieces of sub-analysis. I would suggest having more subheadings to help segment the text into the separate pieces of sub-analysis. I also would ask the authors to consider having results subheadings be descriptive of the main finding of that portion of the analysis (e.g. one example format can be "Sensitivity analysis: Short statement of key finding added here") That would give the reader an early indication of the key messages to be taken away, to be later reinforced by what is reported in the results text.

Isolation bed capacity: It was not until the discussion that the 2024-target capacity for the number of negative-pressure isolation beds in the ROK was mentioned, at 3500 beds. As a healthcare capacity metric, it would be helpful to have that marked on relevant plots, to then enable the reader to visually see the scenarios where that capacity is breached (or gets very close to maximum capacity).

Tables 3-5: I am unsure what the authors wanted me to take away as the key observations from these tables. L248-250 currently only signpost these tables but do

not provide any comment on the results within them – "the results for different initial numbers and logistic growth rates for vaccinations and the varying effects of social distancing on contact reduction are listed in Tables 3–5." My request to the authors is that they provide comment on the results in these tables.

Tables 3-5: Another item related to these tables is that it may be worthwhile having a figure to convey these results, which may help the reader onboard the information. The tables can then be retained as supporting information. As there are four variables of interest – (i) outcome measures (confirmed cases, deaths, peak severe cases); (ii) % reduction in contacts due to social distancing, (iii) initial vaccination number, (iv) growth rate – one visualisation suggestion is the following:

- Separate figure per outcome measure.
- Then each figure can have multiple panels:
    - Global y-axis of % reduction in contacts due to social distancing
    - Global x-axis of growth rate
    - Then on each panel, multiple raincloud plots with one per initial vaccination number scenario.
- Note that the authors may like to switch around the variables used on the global axes and presented in each panel, to see what visually is strongest for conveying the message the authors would like to convey to the reader.

Credible intervals: I would typically associate this terminology with representing the plausibility/uncertainty of parameter values obtained by fitting the model to available data using Bayesian inference. Rather than describing the ranges of the model simulation projections as credible intervals, my view is it would therefore be more appropriate to instead describe them as "Prediction intervals".

Discussion

Findings in context of previous modelling study results: In its present form I did not find the discussion conveyed the similarities and differences between the findings from this study and findings presented in the existing literature. I suggest to the authors that such details are added to help inform the reader how the findings from this study compare to the previous modelling investigations.

Figures

Fig. 1: To the "vaccinated-exposed" and "vaccinated-infectious" labelled compartments, I suggest also adding the appropriate "identified" or "unidentified" to the compartment label. I acknowledge this would make the compartment label longer than the others in the schematic, but I feel that is information that is worth explicitly stating (especially as that information is explicitly stated for the non-vaccinated exposed and infectious compartments).

Fig.3: Raincloud plot. To combine an illustration of the probability density at different values along with jittered raw data. This can further be supplemented by adding boxplots or other standard measures of central tendency and error. For examples, see figures 3 and 4 of "Raincloud plots: a multi-platform tool for robust data visualization" https://doi.org/10.12688/wellcomeopenres.15191.2

Fig. 6: To help the reader distinguish between the multiple line profiles, as well as the different line colours I would suggest having distinct line styles for each line. Identifying between the different scenarios could also be aided by adding to some of the lines different marker styles.

Fig. 7: I am unsure what the solid and dashed lines represent as the main text states two different associations for the solid lines and none for the dashed lines: “Solid curves indicate the values for cumulative confirmed cases, whereas solid curves indicate the values for cumulative deaths.” Secondly, the plot is very busy with that amount of line profiles on a single panel. My suggestion would be to make the figure a two-panel figure, with one panel showing the PRCC values for cumulative confirmed cases, and the other panel showing the PRCC values for cumulative deaths.

Figure captions: Recommend these are expanded to provide sufficient details so the features of each figure can be understood by the reader without having to refer to the main text. To give a couple of examples, I would suggest removing from the main text and instead placing as part of the relevant figure caption: L222-226 describing Figure 2, instead have as part of the Figure 2 caption; L322-324 describing Figure 7, instead have as part of the Figure 7 caption. Similar changes can be made to the remaining figure captions.
