# Supplementary material for: Smallpox outbreak scenarios and reactive intervention protocols: A mathematical model-based analysis applied to the Republic of Korea
Source: PLoS One. 2026 Feb 10;21(2):e0315072. doi: 10.1371/journal.pone.0315072 (PMC12890121; doi:10.1371/journal.pone.0315072)
Supplement: Attachment — Submitted filename: CommentsToAuthor_PONE-D-24-53297_R1.pdf [file pone.0315072.s003.pdf]

Comments to the Authors

Manuscript ID: PONE-D-24-53297_R1

I have reviewed this manuscript from the perspective of a mathematical epidemiologist who has experience in infectious disease modelling, though with no prior experience in modelling the transmission dynamics of smallpox.

This submission is a revision of a study that presents a mathematical model to analyse potential smallpox epidemics, considering factors like age, contact patterns and intervention strategies (contact tracing, targeted vaccination and mass vaccination).

I appreciate the care the authors have taken to address reviewer comments. The provision of open code, additional explanation of the results and expanded motivation of the modelling study are welcomed.

Major comments

I outline below what are, in my opinion, remaining items to undergo further consideration.

Code repository
It is great that the code is now available in a public repository. Thank you to the authors for providing that. I was able to run the main file without issues in MATLAB. I do have the following comments that if addressed would further improve the reproducibility of the work.

- I did not see any documentation provided with the repository to outline what the purpose of each file was. Addition of a README type file that provides that information I think would be helpful. That can also state the version of MATLAB used for the study and any prerequisite packages needed to run the study code.
- There is a lack of comments in each code script. That makes it challenging to understand the purpose of blocks of code. A suggestion to add comments to the code scripts to help guide the user.
- File 'scp_plot_check.m': Ran without error for me as long as I had first run the file 'scp_sto_main.m'. However, if running the file with a clear workspace, the process errored as the prerequisite variables had not been generated. Please can amendments be made to avoid that situation (e.g. Another MAT file can be loaded that has example data to produce the plot set)?
- File 'scpaux_tauleap_v1.m': Also got errors attempting to run this file, even after running the main script (specifically, programme terminated on line 31 as the variable 'scpaux_tauleap_v1' had not been defined). Please check and provide extra information on how this file should be used by the user.

Methods: Parameterisation of social contacts, close contacts and transmission risk
For reproducibility purposes and to permit verification of model applicability, I believe more information needs to be provided on the parameterisation of social contacts, close contacts and transmission risk in the model.

I consider it reasonable that the main text reports the parameter values used. i.e. Overall basic reproduction number of six. Probability of successful disease transmission through close contact set to 60% and through social contact set to 10%. Social contacts occur at four times the frequency of close contacts.

However, I think what is then also needed is an expanded mathematical description as a section in the Supporting Information to describe the calibration of the close contact and social contact contributions to the overall reproduction number. i.e. Given "the basic

reproductive number through close contact was calculated to be approximately four", how the social contact parameters set at the stated relative contact frequency (compared to close contacts) and the successful disease transmission through social contact values results in the contribution of social contacts to the overall basic reproduction number being two.

Methods: Sensitivity analysis
Helpful information has now been added into the revised manuscript to describe the sensitivity analysis. That information has all been added into the Results section. In my opinion, a substantial part of that added text is methods (L410-437). For ease of reader reference, the text describing the methodology for conducting the sensitivity analysis I would consider to be better placed at the end of the Methods section. The results subsection on the sensitivity analysis can then instead open with a high-level summary/reminder to the reader of what the purpose of the sensitivity analysis is in the context of this study.

Figure captions
I provided the following comment in my original review: "Recommend these are expanded to provide sufficient details so the features of each figure can be understood by the reader without having to refer to the main text. To give a couple of examples, I would suggest removing from the main text and instead placing as part of the relevant figure caption: L222-226 describing Figure 2, instead have as part of the Figure 2 caption; L322-324 describing Figure 7, instead have as part of the Figure 7 caption. Similar changes can be made to the remaining figure captions."

The authors have actioned the couple of examples I suggested. However, I have not seen similar edits made to the other figure captions. I still view that for the other figure captions it would be helpful for more information to be given (for purpose of enabling the features of each figure to be understood by the reader without having to refer to the main text).

Figure 6
This new figure helpfully summarises variability in outcomes under different assumptions. To enable the reader to see the underlying outcome distributions, a request to please change the box plots to be raincloud plots.

Figure 9 (formerly Figure 6)
The authors have made edits to help distinguish the different line profiles from one another. These have helped, though I think further changes would be useful to make clear to the reader the differences in outcomes between the different scenarios.

- The age-based order scenarios are both solid lines. The not age-based order scenarios are both dashed lines. To help the reader distinguish between the lines in these respective pairs, a suggestion to add different marker styles to the lines (e.g. crosses, filled circles, unfilled circles, triangles)
- Could present the daily case numbers and severe patient numbers on a log scale
- Adding two additional panels to the figure that show the difference between each scenario compared to the baseline strategy.
    - Panel C showing the differences compared to the baseline strategy for 'Daily confirmed cases'
    - Panel D showing the differences compared to the baseline strategy for 'Severe patients'

Figure 10 (formerly Figure 7)
The split of this figure into two separate panels I found has made the overall figure less busy. I do have other concerns regarding accessibility, due to the line styles all being the same and some of the colours being similar. I recommend edits are made to help the reader distinguish between the different line profiles. One suggestion is to use a monochromatic colour scheme – a single colour for all the line profiles with each line profile having a

different shading intensity (so that across the scenarios the line profiles range from a light shade to a dark shade).

Minor comments

Please find below additional line-by-line comments, which I think would help enhance the quality and clarity of the manuscript.

Abstract

L29-30: “This study develops a mathematical model to evaluate outbreak scenarios and the effectiveness of reactive intervention strategies in controlling transmission.” Please add that this evaluation is applied to the Republic of Korea.

Introduction

L95: “negative-pressure isolation beds to 3,500 to response to Disease-X”. Amend “response” to “respond”.

Methods

L244: Please add the version number of MATLAB that the study code was run with.

Results

The suggested subheadings listed in the Response to Reviewers I view as sensible. However, the subheadings in the revised text appear to slightly differ. I suggest amending them to match the list below stated by the authors in the Response to Reviewers document:

- Baseline Scenario Simulation: Outbreak Outcomes
- Baseline Scenario Simulation: Impact of Outbreak Recognition on Outbreak Size
- Baseline Scenario Simulation: Vaccine Administration Over Time
- Sensitivity Analysis: Factors with Changing Influence Over Time

Figures & Tables

Table 1: A suggestion to add to the table caption that for the sensitivity analysis, a +/- 25% variability around baseline values was applied.

Tables 2-4 (formerly Tables 3-5): As raised in my original review and by Reviewer 3, to streamline the main text these tables can be placed into Supporting Information. The reader now has Figure 6 as the main visual to analyse the results. If the reader were interested in the exact numbers, then can be signposted within the main text to look at the tables.

Figure 4: A comment from Reviewer 3 requested that ‘phases 1-3’ be highlighted on the figure. I did not see that detail added in the revised manuscript. I agree with Reviewer 3 that such an inclusion would provide beneficial contextual information to the reader. I therefore also request that detail is added.

Figure 5: I thank the authors for having revised the plot style to violin plots (from box plots), which I consider to be more informative due to displaying to the reader the probability density. As suggested in my original comment, I think this plot would be further enhanced being presented as a raincloud plots*, due to a raincloud plot displaying as a combination both the probability density at different values along with jittered raw data.

*For examples of raincloud plots, see figures 3 and 4 of “Raincloud plots: a multi-platform tool for robust data visualization” https://doi.org/10.12688/wellcomeopenres.15191.2
