# Supplementary material for: Smallpox outbreak scenarios and reactive intervention protocols: A mathematical model-based analysis applied to the Republic of Korea
Source: PLoS One. 2026 Feb 10;21(2):e0315072. doi: 10.1371/journal.pone.0315072 (PMC12890121; doi:10.1371/journal.pone.0315072)
Supplement: Attachment — Submitted filename: Response to reviewers.docx [file pone.0315072.s004.docx]

**Reviewer #1:**

**Comment 1-1.**

This study constructs a mathematical model for epidemic simulation, which provides a new reference for the prediction and response of smallpox epidemics under the background of bioterrorism. The overall logic is reasonable, the argument is clear, and the conclusion is relatively reliable. It is recommended to add a brief introduction to the existing relevant mathematical models and the innovation of this model in the introduction section.

**Response 1-1.**

Thank you for your positive evaluation of our study. As per your suggestion, we have added an introduction to existing mathematical modeling studies relevant to smallpox and epidemic control (L109-120):

*Recent studies have expanded the scope of smallpox modeling, addressing various contexts and control strategies. Mohanty evaluated the impact of a hypothetical smallpox attack in India and assessed the effectiveness of different control measures, highlighting the importance of rapid response strategies in densely populated regions (13). Similarly, Costantino investigated optimal vaccination strategies during a smallpox outbreak linked to bioterrorism, emphasizing the role of targeted vaccination campaigns in mitigating outbreak severity (14). Several mathematical modeling studies have demonstrated the effectiveness of these interventions in mitigating disease spread. For instance, Rai analyzed the impact of social media advertisements on COVID-19 transmission dynamics, showing that public awareness campaigns significantly influenced behavioral changes and reduced infection rates [15]. Similarly, Sarkar developed a mathematical model to forecast COVID-19 transmission and demonstrated how social distancing and contact tracing played a crucial role in reducing case numbers across different regions [16].*

**Reviewer #2:**

**Comment 2-1.**

Open code: There was no link given to a code repository associated with the study. To enable replication by others, I would strongly encourage that a publicly accessible code repository is made containing the model simulation scripts and scripts to reproduce the figures presented in the manuscript.

**Response 2-1.**

Thank you for your suggestion. In response to your comment, we created a publicly accessible code (MATLAB) repository containing the model simulation scripts and the scripts used to reproduce the figures presented in the manuscript. The repository can be accessed at https://doi.org/10.6084/m9.figshare.28329263.v1.

***The data availability***

*The data that support the findings of this study are openly available in figshare at https://doi.org/10.6084/m9.figshare.28329263.v1*

**===================================================================================**

**Comment 2-2.**

Title: I suggest that the title is amended to reflect that the modelling work is applied in the context of the Republic of Korea. E.g. Smallpox bioterrorism scenarios and reactive intervention protocol: mathematical model-based analysis applied to the Republic of Korea.

**Response 2-2.**

Thank you for your suggestion regarding the title. Following your recommendation, we amended the title to explicitly reflect that the modeling work was applied in the context of the Republic of Korea.

The revised title was updated as follows:

*Smallpox bioterrorism scenarios and reactive intervention protocols: A mathematical model-based analysis applied to the Republic of Korea*

**===================================================================================**

**Comment 2-3.**

The current L344-345 in the discussion – “By 2024, the ROK plans to expand the number of negative-pressure isolation beds to 3500 to respond to emerging infectious diseases” – I would deem as relevant contextual information to provide the reader within the Introduction.

**Response 2-3.**

Thank you for your suggestion. We agree that the context regarding the expansion of negative-pressure isolation beds in the Republic of Korea (ROK) is relevant background information that would help frame the study for readers. To address this, we have relocated this information to the Introduction and revised the text to provide a broader public health preparedness context (L93-95):

*In recognition of the importance of healthcare system readiness, the Republic of Korea (ROK) has planned to expand its capacity for negative-pressure isolation beds to 3,500 [9]. This expansion aims to strengthen the country's ability to respond to emerging infectious disease threats, ensuring that adequate hospital resources are available for managing highly transmissible and severe infections such as smallpox in the event of a bioterrorism attack.*

**Comment 2-4.**

References to previous modelling studies: These were all from before 2010. There appear to be more recent modelling studies that the authors may consider adding to the paragraph on previous modelling studies (L81-91):

B Mohanty et al. (2020). Modelling the impact of a smallpox attack in India and influence of disease control measures. BMJ open, 10(12), e038480. https://doi.org/10.1136/bmjopen-2020-038480

V. Costantino, M. Kunasekaran & C.R. MacIntyre. (2021). Modelling of optimal vaccination strategies in response to a bioterrorism associated smallpox outbreak. Human Vaccines & Immunotherapeutics, 17(3), 738-746. https://doi.org/10.1080/21645515.2020.1800324

**Response 2-4.**

Thank you for pointing out the importance of including more recent studies on smallpox modeling. We have revised the paragraph on previous modeling studies to include references to the work by Mohanty et al. (2020) and Costantino et al. (2021). These studies provided valuable insights into the effectiveness of control measures and vaccination strategies in different geographic and demographic contexts, further reinforcing the relevance of our research. Following is the revised part in the manuscript L109.

*Recent studies have expanded the scope of smallpox modeling, addressing various contexts and control strategies. Mohanty evaluated the impact of a hypothetical smallpox attack in India and assessed the effectiveness of different control measures, highlighting the importance of rapid response strategies in densely populated regions. Similarly, Costantino investigated optimal vaccination strategies during a smallpox outbreak linked to bioterrorism, emphasizing the role of targeted vaccination campaigns in mitigating outbreak severity.*

**===================================================================================**

**Comment 2-5.**

Contact structure: The reader is currently directed to a supporting reference for the age-group contact data. Given the assumptions being made between the close contacts and social contacts, I personally would find it very helpful to have a two-panel figure visualising the age-group contact structure, with one panel showing the values used for close contacts and the other panel the values used for social contacts.

**Response 2-5.**

Thank you for the suggestion to include a visual representation of the age-group contact structures. We agree that such a figure would enhance the clarity of our manuscript. To address this, we have added a two-panel heatmap figure (Figure 2) showing the contact matrices used in our study. The first panel represents the close contact structure, and the second panel represents the social (casual) contact structure.

**Comment 2-6.**

Sensitivity analysis: For reproducibility purposes, a description of the sensitivity analysis conducted should be added to the Methods (including moving what is currently expressed on L312-321 from the Results to the Methods). I recommend the range of values considered for the parameter sensitivity analysis to also be added to Table 1, along with citing additional references as necessary to support the stated parameter value ranges.

**Response 2-6.**

Thank you for emphasizing the need to provide a detailed description of the sensitivity analysis in the Methods section for reproducibility. In this study, we simply applied *±*25% range for each parameter. In response, we have made the following changes (L434):

*Latin hypercube sampling was used to generate samples from parameter ranges informed by ±25% variability around baseline values. PRCC values were calculated over time to evaluate the relative importance of each parameter in influencing the model outputs.*

**===================================================================================**

**Comment 2-7.**

Programming language and computing resources information: What was the programming language, and its version, used to perform the model simulations? Were there any additional packages that were used? Please add this information as part of the methods. Please also state if any high-performance computing resource was used for this modelling study.

**Response 2-7.**

We used MATLAB for this study, and we included these state in L244. In this study, we made the code for this study and uploaded the simulation code in the depository (https://doi.org/10.6084/m9.figshare.28329263.v1).

**===================================================================================**

**Comment 2-8.**

L110-111: The text states: “we categorized the population into 16 age groups, each spanning 5 years, ranging from 0–4 years to 65 years and older in the Republic of Korea”. However, that age range would only give 14 age groups. Table 2 later shows 5-year age groups up to 75 years and older. Please revise the text to correspond with the information in Table 2.

**Response 2-8.**

Corrected (65 -> 75).

**Comment 2-9.**

Table 2: I suggest this is turned into a figure. For example, a horizontal bar plot with a bar for each age group. The value can then still be stated on the figure, potentially within the bar itself or to the right-hand side of the bar.

**Response 2-9.**

Thank you for your suggestion to convert Table 2 into a figure. Following your recommendation, we have replaced Table 2 with a figure (Figure 3), which displays a horizontal bar plot representing the population distribution across age groups.

Figure 3. Age-group population distribution used in the model. The horizontal bar graph represents the population size for each age group. The numbers above the bars indicate the population size in millions (e.g., 3.5 represents 3.5 million people).

**===================================================================================**

**Comment 2-10.**

Subheadings: The baseline scenario results section contains multiple figures and multiple pieces of sub-analysis. I would suggest having more subheadings to help segment the text into the separate pieces of sub-analysis. I also would ask the authors to consider having results subheadings be descriptive of the main finding of that portion of the analysis (e.g. one example format can be “Sensitivity analysis: Short statement of key finding added here”) That would give the reader an early indication of the key messages to be taken away, to be later reinforced by what is reported in the results text.

**Response 2-10.**

We have added and revised the following subheadings to improve clarity and structure:

- Baseline Scenario Simulation: Outbreak Outcomes
- Baseline Scenario Simulation: Impact of Outbreak Recognition on Outbreak Size
- Baseline Scenario Simulation: Vaccine Administration Over Time
- Sensitivity Analysis: Factors with Changing Influence Over Time

**Comment 2-11.**

Isolation bed capacity: It was not until the discussion that the 2024-target capacity for the number of negative-pressure isolation beds in the ROK was mentioned, at 3500 beds. As a healthcare capacity metric, it would be helpful to have that marked on relevant plots, to then enable the reader to visually see the scenarios where that capacity is breached (or gets very close to maximum capacity).

**Response 2-11.**

The 3500-bed capacity refers the beds for severe cases. While this capacity is relevant to the blue curve in Figure 4, the specific 3500-bed threshold is associated with the dashed line representing severe cases, not the solid line representing non-severe cases. We initially chose not to mark this capacity on the plot to avoid potential confusion, as it does not apply to the non-severe cases. Instead, we addressed this point explicitly in the main text to clarify its relevance.

**===================================================================================**

**Comment 2-12.**

Tables 3-5: I am unsure what the authors wanted me to take away as the key observations from these tables. L248-250 currently only signpost these tables but do not provide any comment on the results within them – “the results for different initial numbers and logistic growth rates for vaccinations and the varying effects of social distancing on contact reduction are listed in Tables 3–5.” My request to the authors is that they provide comment on the results in these tables.

**Response 2-12.**

We revised the result part (L333) as follows:

In addition to the baseline scenario, Tables 2–4 present the outbreak outcomes under different initial vaccination numbers, vaccination growth rates, and levels of social distancing. When contact reduction increased from 50% to 70%, total confirmed cases decreased substantially, from 90,947 to 17,565 under the baseline vaccination scenario (1,000 initial vaccinations, growth rate = 0.1). Similarly, total deaths declined from 11,890 to 2,851. Faster vaccine deployment also had a significant impact; for instance, under 50% contact reduction, increasing the vaccination growth rate from 0.05 to 0.5 reduced confirmed cases from 260,458 to 29,323 and deaths from 34,255 to 3,816.

**Comment 2-13.**

Tables 3-5: Another item related to these tables is that it may be worthwhile having a figure to convey these results, which may help the reader onboard the information. The tables can then be retained as supporting information. As there are four variables of interest: Outcome measures (confirmed cases, deaths, peak severe cases), % reduction in contacts due to social distancing, Initial vaccination number, Growth rate - One visualisation suggestion is the following:

- Separate figure per outcome measure.
- Then each figure can have multiple panels:
  - Global y-axis of % reduction in contacts due to social distancing
  - Global x-axis of growth rate
  - Then on each panel, multiple raincloud plots with one per initial vaccination number scenario.
- Note that the authors may like to switch around the variables used on the global axes and presented in each panel, to see what visually is strongest for conveying the message the authors would like to convey to the reader.

**Response 2-13.**

Thank you for your helpful suggestion. However, as both the x- and y-axes represent model input variables, it was not feasible to implement the raincloud plot as requested. Instead, we visualized the results using box-and-whisker plots, with the input variables represented through x-axis positioning and color grouping. Please note that raincloud plot was not feasible as the scale of output was relatively huge (log-scale in visualization). The manuscript has been revised accordingly to reflect this change (L333):

*In addition to the baseline scenario, Figure 6 visualizes the distribution of outbreak outcomes according to the vaccination growth rate and the reduction in contacts due to social distancing, assuming an initial vaccination number of 1,000.*

*Figure 6. Outbreak outcomes by vaccination growth rate and contact reduction under the baseline initial vaccination number (1,000) in log scale: Confirmed cases (A), deaths (B), and peak severe patients (C). Colors represent vaccination growth rates.*

**Comment 2-14.**

Credible intervals: I would typically associate this terminology with representing the plausibility/uncertainty of parameter values obtained by fitting the model to available data using Bayesian inference. Rather than describing the ranges of the model simulation projections as credible intervals, my view is it would therefore be more appropriate to instead describe them as “Prediction intervals”.

**Response 2-14.**

We corrected Credible interval to Prediction interval.

**===================================================================================**

**Comment 2-15.**

Findings in context of previous modelling study results: In its present form I did not find the discussion conveyed the similarities and differences between the findings from this study and findings presented in the existing literature. I suggest to the authors that such details are added to help inform the reader how the findings from this study compare to the previous modelling investigations.

**Response 2-15.**

We have added a paragraph in the Discussion comparing our findings with previous smallpox modeling studies. This section highlights key similarities, such as the importance of early detection, NPIs, and vaccination, while also noting novel aspects of our study, including vaccine prioritization strategies and healthcare capacity considerations (L457):

*Our findings align with previous modeling studies emphasizing early outbreak recognition and rapid intervention as key factors in controlling smallpox outbreaks. Ferguson et al. and Meltzer et al. demonstrated the effectiveness of ring vaccination and quarantine measures, which our results support by showing that strong NPIs combined with rapid vaccination significantly reduce outbreak severity.*

*Unlike prior studies that focused on single vaccination strategies, our study evaluates different vaccine rollout speeds and prioritization methods. While Mohanty et al. and Costantino et al. highlighted the role of vaccination in outbreak control, our results suggest that prioritizing high-transmission age groups is more effective than prioritizing high-risk older adults, particularly in scenarios with limited NPIs. Additionally, our study expands upon Ohkusa et al. by showing that mass vaccination is essential when outbreak recognition is delayed, reinforcing the importance of both proactive and reactive strategies. By incorporating Korea-specific demographic data, healthcare capacity constraints, and a logistic vaccine administration model, our analysis provides a more context-specific policy recommendation while contributing to broader smallpox preparedness research.*

**Comment 2-16.**

Fig. 1: To the “vaccinated-exposed” and “vaccinated-infectious” labelled compartments, I suggest also adding the appropriate “identified” or “unidentified” to the compartment label. I acknowledge this would make the compartment label longer than the others in the schematic, but I feel that is information that is worth explicitly stating (especially as that information is explicitly stated for the non-vaccinated exposed and infectious compartments).

**Response 2-16.**

Corrected based on your comment as follows:

**Comment 2-17.**

Fig. 3: Raincloud plot. To combine an illustration of the probability density at different values along with jittered raw data. This can further be supplemented by adding boxplots or other standard measures of central tendency and error. For examples, see figures 3 and 4 of “Raincloud plots: a multi-platform tool for robust data visualization” <https://doi.org/10.12688/wellcomeopenres.15191.2>

**Response 2-17.**

Thank you for your suggestion. We revised the figure as follows:

**Comment 2-18.**

Fig. 6: To help the reader distinguish between the multiple line profiles, as well as the different line colours I would suggest having distinct line styles for each line. Identifying between the different scenarios could also be aided by adding to some of the lines different marker styles.

**Response 2-18.**

To distinguish scenarios, we have revised the figure by using dashed (not age-based order) and dotted lines (baseline scenario) as follows:

**Comment 2-19.**

Fig. 7: I am unsure what the solid and dashed lines represent as the main text states two different associations for the solid lines and none for the dashed lines: “Solid curves indicate the values for cumulative confirmed cases, whereas solid curves indicate the values for cumulative deaths.” Secondly, the plot is very busy with that amount of line profiles on a single panel. My suggestion would be to make the figure a two-panel figure, with one panel showing the PRCC values for cumulative confirmed cases, and the other panel showing the PRCC values for cumulative deaths.

**Response 2-19.**

As your suggestion, we separated the figure into two panels.

**Comment 2-20.**

Figure captions: Recommend these are expanded to provide sufficient details so the features of each figure can be understood by the reader without having to refer to the main text. To give a couple of examples, I would suggest removing from the main text and instead placing as part of the relevant figure caption:

L222-226 describing Figure 2, instead have as part of the Figure 2 caption

L322-324 describing Figure 7, instead have as part of the Figure 7 caption. Similar changes can be made to the remaining figure captions.

**Response 2-20.**

We revised as your suggestions:

Figure 2 (now 4) caption

*Figure 4. Baseline scenario simulation results. The curves represent the mean values of the model simulations, and shaded areas indicate the 95% prediction interval. The red graph shows the daily confirmed cases, whereas the blue curves represent isolated patients. Among the blue curves, the solid and dashed lines indicate patients with non-severe and severe patients.*

Figure 7 (now 9) caption

*Figure 9. Absolute value of PRCC over time. The colors in the graph represent the model inputs. Solid curves indicate the values for cumulative confirmed cases, whereas solid curves indicate the values for cumulative deaths.*

**Reviewer #3:**

**Comment 3-1.**

This manuscript is a simulated study based on the SIR model. It uses a mathematical model to analyze the smallpox epidemic, including the control measures. The topic is not interesting for public health since this disease has not been an outbreak since 1980. The introduction should be more convincing as to why the authors chose smallpox in this study. The evidence of small outbreaks may help to improve the introduction. The authors should be concerned about using some specific words without definitions, such as cases, patients, isolated patients, ring or mass vaccinations, and confirmed cases. These words have their definitions. The intervention strategies are not clear on how to apply them.

**Response 3-1.**

We revised the manuscript as follows (L77):

*In recent years, additional laboratory-related incidents have raised concerns about smallpox security. In 2014, live smallpox vials were accidentally discovered at the U.S. Centers for Disease Control and Prevention (CDC), and in 2021, unapproved vials labeled "VARIOLA" were unexpectedly found in a vaccine research facility in Pennsylvania. Furthermore, advancements in synthetic biology have led to concerns about the feasibility of reconstructing the variola virus. In 2017, scientists successfully synthesized the horsepox virus, a close relative of smallpox, highlighting the potential for recreating smallpox through genetic engineering. These incidents illustrate that despite eradication, the risk of smallpox re-emergence remains a global security and public health concern.*

We added following introductive part to describe the definitions of words (L129):

*To enhance clarity, we define key epidemiological terms used in this study: "cases" refer to all infected individuals regardless of symptom severity, "confirmed cases" are those identified through clinical diagnosis or laboratory confirmation, "isolated patients" are confirmed cases placed under strict containment, "ring vaccination" targets close contacts of confirmed cases, and "mass vaccination" refers to large-scale immunization of the general population regardless of exposure history.*

**===================================================================================**

**Comment 3-2.**

What are the meanings of ring or mass vaccinations in this study? The authors used a mathematical model (ODE fashion) via the Tau-leaping method. Normally, ring vaccination is applied for spatial models or for those who have close contact with infected cases via an individual-based model. I would suggest using another word.

**Response 3-2.**

To clarify, we have updated the terminology:

"Ring vaccination" → "Targeted vaccination", as it represents a proactive vaccination strategy applied immediately after case detection.

**Comment 3-3.**

“Using this value and the next-generation matrix method, the basic reproductive number through close contact was calculated to be approximately four (16).” It is not clear how to define close contact or social contact.

**Response 3-3.**

In this study, we set the basic reproductive number (R₀) of smallpox at 6 based on literature review, with a 60% probability of transmission through close contact. When applying the next-generation matrix method to the close contact matrix, the estimated R₀ was approximately 4, suggesting that social contacts contribute approximately 2 to the total R₀. To reflect this, we assumed that social contacts occur at four times the frequency of close contacts and set the probability of transmission through social contact at 10%.

Accordingly, we have revised the manuscript (L176) as follows:

*Using this value and the next-generation matrix method, the basic reproductive number through close contact was calculated to be approximately four (18). Considering that the recorded basic reproductive number for smallpox is around six, this suggests that social contacts contribute about two. To reflect this, we assumed that social contacts occur at four times the frequency of close contacts and set the probability of transmission through social contact at 10%, based on this frequency difference (19).*

**===================================================================================**

**Comment 3-4.**

Please separate the control measures into a section so that they can be understood the results. The vaccination strategies should be clear in this section.

**Response 3-4.**

Thank you for your suggestion to separate the control measures into a dedicated section for clarity. In response, we have added a new “Control Measures” section at the beginning of the Methods (L140) as follows:

***Control measures***

*This study incorporates the following intervention strategies to analyze their impact on smallpox outbreak dynamics. Details on how these intervention strategies are incorporated into the model are provided in the following sections.*

*•Contact Tracing: Identification and isolation of individuals who have had close contact with confirmed cases to prevent further transmission.*

*•Social Distancing: Reduction of overall contact number in the population to limit the spread of infection.*

*•Targeted Vaccination: In response to confirmed cases, a proportion of susceptible individuals in the population is vaccinated to simulate the effect of a containment-focused vaccination strategy. In our ODE-based model, this is implemented by prioritizing a subset of the population for immediate vaccination following outbreak recognition.*

*•Mass Vaccination: A broad immunization campaign administered across the population, independent of exposure history. This strategy is modeled as a gradual increase in vaccination coverage following a logistic growth function, accounting for vaccine distribution constraints.*

**Comment 3-5.**

For “Baseline model simulation scenario” for phase 2, what happens if the duration is more than three days? I think three days is very short to prepare for the next phase.

**Response 3-5.**

Due to the Republic of Korea’s geopolitical circumstances, the country maintains a constant stockpile of smallpox vaccines. The three-day delay was determined not by the modeler, but through consultation with experts from national institutions responsible for vaccine stockpiling and emergency response. Alternatively, to account for uncertainty in vaccine rollout speed, this study includes scenario-based analyses and sensitivity analyses on vaccination rates.

**===================================================================================**

**Comment 3-6.**

“The number of exposed hosts was set to 100 and proportionally distributed according to the population ratio of each group.” How does this set the initial condition, is it constant over 1000 simulations or randomly based on some function, please clarify.

**Response 3-6.**

Thank you for your comment. We clarify that the initial number of exposed hosts was fixed at 100 and distributed proportionally to each age group’s population size so that the total remained constant at 100. This distribution was not randomized but remained the same across all 1000 simulations. Following is revised part (L245).

*As an initial condition for the model simulation, all population groups were assumed to be in a susceptible state. The number of exposed hosts was fixed at 100 and was proportionally distributed among age groups based on their population sizes, ensuring that the total remained constant at 100. This distribution was deterministic and remained the same across all simulations, rather than being randomly assigned. Table 2 lists the population numbers in each group.*

**Comment 3-7.**

Provide more detail on “The age groups with higher transmission risk were calculated based on the contact rate and the probability of successful disease transmission, and the order was as follows: age groups 4 (15-19 year), 3 (10-14 year), 9 (40-44 year), 8 (35-39 year), 7 (30-34 year), 6 (25-29 year), 10 (45-49 year), 11 (50-54 year), 5 (20-24 year), 2 (5-9 year), 12 (55-59 year), 13 (60-64 year), 1 (0-4 year), 14 (65-69 year), 15 (70-74 year), 16 (75+ year).”, the authors may show in the supplement.

**Response 3-7.**

The transmission risk ranking of age groups was determined by calculating the column sum of the next-generation matrix, representing the total potential transmission risk each age group poses to others. We revised the manuscript as follows (L291):

*The age groups with higher transmission risk were determined by calculating the column sum of the next-generation matrix, which represents the total potential transmission risk each age group poses to others. Since the probability of successful disease transmission per close or social contact does not vary by age group in our model, the ranking is proportional to the contact rates and population size, and the order was as follows:*

**===================================================================================**

**Comment 3-8.**

Results: Baseline scenario simulation, please provide the symbol of compartments to make it easy to track the results. I would be more understanding of the baseline simulation if the authors could provide the results of no control.

**Response 3-8.**

We revised the manuscript by providing the symbol of compartments with the results as follows (L301):

*Figure 4 shows the model simulation results for the confirmed cases (*$\sum_{i} \tilde{\alpha}\tilde{I}_{i}+\alpha I_{i}$*, transition from infectious to isolated) and isolated patients (*$\sum_{i} \left[ \tilde{\alpha}\left( \tilde{I}_{j}+\tilde{I}_{j}^{v} \right)+\alpha\left( I_{j}+I_{j}^{v} \right) \right]$*). Daily confirmed cases increased sharply (mean 1167, maximum 1602 in 95% prediction interval; PI) owing to contact tracing implemented at the initial outbreak recognition, and then decreased, followed by a gradual increase, and finally decreased again owing to herd immunity from vaccination. The number of non-severe patients (*$\sum_{i} \left( Q_{i}+Q_{i}^{v} \right)$*) reached a mean of 3750 (maximum 5422 in 95% PI) after 114 days of spread, whereas that of severe patients (*$\sum_{i} \left( H_{i}+H_{i}^{v} \right)$*) reached a mean of 1235 (maximum 1838 in 95% PI) after 99 days of exposure.*

**===================================================================================**

**Comment 3-9.**

Figure 2, please highlight the phases 1-3.

**Response 3-9.**

We revised the figure legend for more self-explanatory as follows:

*Figure 4. Baseline scenario simulation results. The curves represent the mean values of the model simulations, and shaded areas indicate the 95% prediction interval. The red graph shows the daily confirmed cases, whereas the blue curves represent isolated patients. Among the blue curves, the solid and dashed lines indicate patients with non-severe and severe patients.*

**Comment 3-10.**

“In addition to the baseline scenario, the results for different initial numbers and logistic growth rates for vaccinations and the varying effects of social distancing on contact reduction are listed in Tables 3–5.” What do the authors mean by different initial numbers? To be clear, the authors should provide the figure for vaccinations.

**Response 3-10.**

Thank you for your helpful suggestion. We visualized the results using box-and-whisker plots, with the input variables represented through x-axis positioning and color grouping. The manuscript has been revised accordingly to reflect this change (L333):

*In addition to the baseline scenario, Figure 6 visualizes the distribution of outbreak outcomes according to the vaccination growth rate and the reduction in contacts due to social distancing, assuming an initial vaccination number of 1,000.*

*Figure 6. Outbreak outcomes by vaccination growth rate and contact reduction under the baseline initial vaccination number (1,000) in log scale: Confirmed cases (A), deaths (B), and peak severe patients (C). Colors represent vaccination growth rates.*

**Comment 3-11.**

Rewrite “Panels A and B show the confirmed cases, and panels C and D show the number of deaths.” To correspond with the Figure 3.

We revised the paragraph considering your comment as follows (L319):

*Figure 5 presents the distribution of confirmed cases and deaths from the model simulations across age groups using box-and-whisker plots. Panel A shows the total number of confirmed cases across all age groups, while Panel B displays the age-specific distribution of confirmed cases. Similarly, Panel C illustrates the total number of deaths, and Panel D presents the age-specific death distribution. The total number of confirmed cases was 36,600 (95% PI [24,253, 51,500]), and the total number of deaths was 5,345 (95% PI [3,472, 7,545]). Age group 9 (40–44 years) had the highest number of confirmed cases, with a mean of 4,241 (95% PI [2,846, 5,969]), while age group 11 (50–54 years) had the highest number of deaths, with a mean of 677 (95% PI [447, 936]). The 0–4 years age group had the lowest number of confirmed cases (mean 361, 95% PI [237, 513]) and deaths (mean 68, 95% PI [41, 101]).*

**===================================================================================**

**Comment 3-12.**

Tables 3-5 can be combined and presented in one bar plot. Those tables can move to supplementary.

**Response 3-12.**

Thank you for your helpful suggestion. We visualized the results using box-and-whisker plots, with the input variables represented through x-axis positioning and color grouping. The manuscript has been revised accordingly to reflect this change (L333):

*In addition to the baseline scenario, Figure 6 visualizes the distribution of outbreak outcomes according to the vaccination growth rate and the reduction in contacts due to social distancing, assuming an initial vaccination number of 1,000.*

*Figure 6. Outbreak outcomes by vaccination growth rate and contact reduction under the baseline initial vaccination number (1,000) in log scale: Confirmed cases (A), deaths (B), and peak severe patients (C). Colors represent vaccination growth rates.*

**Comment 3-13.**

There is a lag on how to calculate probability. Revise the figure caption to be clearer, i.e., what is D+28.

**Response 3-13.**

We revised the figure x-axis label for self-explanatory

**===================================================================================**

**Comment 3-14.**

Figure 5: it is not clear how to define “the mean daily vaccination number”.

**Response 3-14.**

We clarify that the term "mean daily vaccination number" is used because targeted vaccination numbers fluctuate across simulations, as they depend on the number of detected contacts in each run. In contrast, population-wide vaccination follows a predetermined schedule and does not vary between simulations. We have revised the manuscript to clearly define this distinction (L375).

*The mean daily vaccination number refers to the average number of vaccines administered per day across all simulations, which varies for targeted vaccination due to its dependency on the number of identified contacts in each run. In contrast, population-wide vaccination follows a fixed schedule and remains constant across simulations.*

**===================================================================================**

**Comment 3-15.**

Figure 6: Is it cases or patients?

**Response 3-15.**

As y-axis labels indicate, Panel A shows cases and B shows patients.

**Comment 3-16.**

Vaccine prioritization should be clear in the method.

**Response 3-16.**

To improve clarity, we revised the Methods section to provide a more detailed explanation of vaccine prioritization. Specifically, we explicitly described the four prioritization strategies considered in this study and how they were applied in the model as follows (L280).

*Vaccination was not prioritized in the baseline scenario considered in this study. However, as observed during the COVID-19 pandemic, strategic prioritization of vaccine distribution can be crucial for outbreak control. To explore the impact of different prioritization strategies, we implemented four distinct vaccination prioritization criteria:*

1. *Ascending age – Vaccination is first administered to the youngest age group and progresses sequentially toward older age groups.*
2. *Descending age – Vaccination is first administered to the oldest age group and progresses sequentially toward younger age groups.*
3. *Prioritizing age groups with higher transmission risk*
4. *Prioritizing age groups with higher severity/death risk – Vaccination is allocated first to age groups with the highest probability of severe disease and mortality.*

**===================================================================================**

**Comment 3-17.**

For PRCC, the authors should provide more detail of each parameter’s link with the mathematical model.

**Response 3-17.**

As you commented, we revised as follows (L417):

*We considered the following model inputs:*

- *Outbreak recognition timing – This parameter determines when interventions begin by setting the delay between the initial exposure and the implementation of control measures. A shorter recognition time allows for earlier contact tracing, isolation, and vaccination.*
- *Impact of social distancing on contact number – This represents the effectiveness of social distancing measures in reducing population-wide contact rates. Higher values indicate greater reductions in interpersonal interactions, which directly affect the force of infection in the model by lowering transmission opportunities.*
- *Infectious period of traced and non-traced cases – These parameters define the duration of infectiousness before an individual is isolated. Traced cases (identified through contact tracing) have a shorter infectious period due to faster isolation, whereas non-traced cases remain infectious for a longer duration, increasing the likelihood of further transmission.*
- *Contact identification ratio – This parameter determines the proportion of an infected individual's contacts that are successfully traced and quarantined. Higher values enhance the effectiveness of contact tracing, reducing the number of secondary infections.*
- *Logistic growth rate of the daily vaccination number – This parameter governs the rate at which vaccination capacity scales up over time. A higher growth rate enables faster vaccine distribution, accelerating immunity buildup and controlling the outbreak more efficiently.*

**Comment 3-18.**

Lines 360-367 could not be understood without the clear methods.

We added extra description in methods section related with the paragraph you mentioned (L198):

*Targeted vaccination was modeled and applied following outbreak recognition vaccination strategy applied following outbreak recognition. The number of individuals requiring immediate vaccination was determined based on the distribution of contagious contacts at the outbreak recognition time point.*

We also revised the discussion session as follows (L488):

*Figure 4 represents the estimated number of individuals requiring immediate vaccination following outbreak recognition, serving as the primary basis for determining the minimum vaccination targets. Given that new exposures continue to occur after outbreak recognition, the additional required vaccinations are illustrated in Figure 5, which captures the cumulative vaccination scope over time. Although these targeted vaccinations constituted only ~0.1% of the total vaccination campaign, they were concentrated in the first week to ensure that the most at-risk individuals received timely immunization. This approach aligns with real-world outbreak control strategies, where early vaccination of high-risk contacts is essential to prevent further spread. The results of this study provide a quantitative framework for determining vaccination distribution points and operational strategies during the early containment phase.*

**===================================================================================**

**Comment 3-19.**

What the authors mean by this “However, if the effective reproduction number increases”, which parameters increase?

**Response 3-19.**

We revised the paragraph as follows for clarity (L503):

*However, if the effective reproduction number increases—for example, due to a higher contact rate, an increased probability of successful disease transmission, or a lower isolation rate of infectious individuals—prioritizing the elderly would be less effective than vaccinating younger adults in minimizing deaths.*

**Comment 3-20.**

Lines 374-381, there is a lot of work doing vaccine prioritization using COVID-19 as a case study. The authors can discuss more on this issue.

**Response 3-20.**

Thank you for valuable comment. We added following paragraphs for further discussion (L513):

*Several studies have emphasized the importance of prioritizing populations based on age, occupation, and health status to achieve the greatest public health impact. For instance, the UK's Joint Committee on Vaccination and Immunisation recommended prioritizing older adults, frontline health and social care workers, and individuals with underlying health conditions during the initial vaccine rollout. This approach aimed to protect those at the highest risk of severe disease while maintaining healthcare system functionality. Similarly, Canada's National Advisory Committee on Immunization advised prioritizing residents and staff of long-term care facilities, adults aged 70 and older, and frontline healthcare workers to reduce COVID-19-related morbidity and mortality by focusing on the most vulnerable populations.*

*These prioritization frameworks underscore the critical role of targeted vaccination strategies in controlling the spread of infectious diseases and minimizing adverse outcomes. They also highlight the importance of adapting vaccination plans based on the epidemiological and demographic context of a given outbreak. While COVID-19 vaccine prioritization largely focused on protecting high-risk individuals, smallpox, with its higher basic reproductive number and different transmission characteristics, may require a different prioritization strategy. Our findings suggest that in the case of smallpox, prioritizing groups with higher transmission rates rather than solely focusing on vulnerable populations could be more effective in reducing overall mortality.*

**Reviewer #4:**

**Comment 4-1.**

The abstract is a little thin and does not quite convey the vibrancy of the findings and the depth of the main conclusions. The authors should please extend this somewhat for a better first impression.

**Response 4-3.**

We revised the abstract as follows:

*Smallpox, caused by the variola virus, remains a potential bioterrorism threat despite its eradication. This study develops a mathematical model to evaluate outbreak scenarios and the effectiveness of reactive intervention strategies in controlling transmission. The model incorporates age-stratified contact patterns, contact tracing, and vaccination strategies, including targeted vaccination and mass vaccination.*

*Our simulations demonstrate that early outbreak recognition and rapid intervention are critical in mitigating smallpox spread. In scenarios where vaccination rollout was slow or outbreak recognition was delayed, severe patient numbers exceeded healthcare capacity, highlighting the need for preemptive preparedness. Sensitivity analyses revealed that outbreak recognition timing and contact tracing effectiveness were the most influential factors in determining outbreak severity, with later recognition leading to up to 3.5 times more cumulative cases.*

*Furthermore, we compared different vaccination prioritization strategies and found that prioritizing high-transmission age groups was more effective in reducing total mortality than prioritizing high-risk groups based solely on disease severity. This contrasts with COVID-19 vaccination strategies, which focused on protecting vulnerable populations.*

*These findings underscore the importance of early detection, strategic vaccination, and non-pharmaceutical interventions in mitigating a potential smallpox outbreak. Our model provides a quantitative framework for policymakers to evaluate intervention effectiveness and optimize outbreak response strategies.*

**===================================================================================**

**Comment 4-2.**

The manuscript lacks motivation. Author needs to include the motivation of the study.

**Response 4-2.**

To emphasize the motivation of the study more clearly, we have added a sentence highlighting recent exposure incidents related to smallpox (L78):

*In recent years, additional laboratory-related incidents have raised concerns about smallpox security. In 2014, live smallpox vials were accidentally discovered at the U.S. Centers for Disease Control and Prevention (CDC), and in 2021, unapproved vials labeled "VARIOLA" were unexpectedly found in a vaccine research facility in Pennsylvania. Furthermore, advancements in synthetic biology have led to concerns about the feasibility of reconstructing the variola virus. In 2017, scientists successfully synthesized the horsepox virus, a close relative of smallpox, highlighting the potential for recreating smallpox through genetic engineering. These incidents illustrate that despite eradication, the risk of smallpox re-emergence remains a global security and public health concern.*

**Comment 4-3.**

To stop the spread of the diseases vaccine is needed. But, in absence of the vaccine people must have maintain the social distancing. In order to maintain the social distancing must obey the modeling rule. The introduction need to be improved by incorporating some recent references of COVID-19 pandemic. To do so, I suggest some modeling work must be included in the references: "Modeling and forecasting the COVID-19 pandemic in India, Chaos, Soliton & Fract. 139 (2020) 110049", “Mathematical modeling of the COVID-19 outbreak with intervention strategies, Results in Physics, 2021, 104285”, "Forecasting the daily and cumulative number of cases for the COVID-19 pandemic in India, Chaos, 30(7) (2020) 071101", "A mathematical model for COVID-19 transmission dynamics with a case study of India, Chaos, Soliton & Fract. 140 (2020) 110173".

In this context an important factor must be include in this study, that is, the impact of the effect of media. How the COVID-19 dynamics has been changed due to incorporation of the media related awareness like use of face masks, non-pharmaceutical interventions, hand sanitization, etc. The authors must include the manuscript, “Impact of social media advertisements on the transmission dynamics of COVID-19 pandemic in India, Journal of Applied Mathematics and Computing (2021)” "Dynamics of the COVID-19 pandemic in India, (2021) arXiv:2005.06286v2." to study the effect of media.

**Response 4-3.**

We have enhanced the Introduction by incorporating recent studies that emphasize the role of non-pharmaceutical interventions (NPIs) and media awareness during the COVID-19 pandemic (L114):

*Several mathematical modeling studies have demonstrated the effectiveness of these interventions in mitigating disease spread. For instance, Rai analyzed the impact of social media advertisements on COVID-19 transmission dynamics, showing that public awareness campaigns significantly influenced behavioral changes and reduced infection rates. Similarly, Sarkar developed a mathematical model to forecast COVID-19 transmission and demonstrated how social distancing and contact tracing played a crucial role in reducing case numbers across different regions.*

**===================================================================================**

**Comment 4-4.**

Is there any experimental data to validate the mathematical model ? The authors at least describe the basic reproduction number R_0 and its impact on covid-19 pandemic. The basic reproduction number R_0 is one of the most crucial quantities in infectious diseases, as R_0 measures how contagious a disease is. For R_0 < 1, the disease is expected to stop spreading, but for R_0 = 1 an infected individual can infect on an average 1 person, that is, the spread of the disease is stable. The disease can spread and become epidemic if R_0 must be greater than 1. In this context the authors include the reference "Mathematical analysis of the global dynamics of a HTLV-I infection model, considering the role of cytotoxic T-lymphocytes, Math. Comput. Simul. 180(2021) 354-378."

**Response 4-4.**

Thank you for your comment. However, the disease considered in this study has a basic reproduction number (R₀) significantly greater than 1 (approximately 6), meaning that scenarios where transmission remains near the threshold of R₀ = 1 are not relevant to our analysis. Our focus is on modeling highly transmissible outbreaks and evaluating intervention strategies in such contexts.

**Comment 4-5.**

Some references contain errors and inconsistent formatting. It is difficult to give credit to research if even elementary aspects of the work are not error free. This should be corrected with care and love to detail.

**Response 4-5.**

Corrected.

**===================================================================================**

**Comment 4-6.**

The manuscript is comprehensive, and I have enjoyed learning about the presented results. I find that the manuscript is written with very poor english and the presentation is not good, and I am in principal in favor of publication, although the following comments should nevertheless be accommodated in one major revision.

**Response 4-6.**

Thank you for your positive feedback on the comprehensiveness of our manuscript and for your thoughtful comments. We would like to clarify that the manuscript has undergone professional English proofreading to ensure clarity and readability. However, we acknowledge your concerns and have carefully addressed all your specific comments in this revision.

To improve the manuscript further, we have:

1. Enhanced the abstract to better highlight the key findings and contributions (Comment 4-1).
2. Expanded the introduction by incorporating recent studies on mathematical modeling and vaccine prioritization during the COVID-19 pandemic (Comment 4-2, 4-3).
3. Clarified the methodological details, particularly regarding intervention strategies, PRCC parameter explanations, and vaccination prioritization criteria (Comments 3-16, 3-17, 3-18).
4. Refined the discussion to provide a clearer comparison between smallpox and COVID-19 vaccination strategies and to emphasize the policy implications of our findings (Comments 4-1, 4-3).
