# Supplementary material for: Smallpox outbreak scenarios and reactive intervention protocols: A mathematical model-based analysis applied to the Republic of Korea
Source: PLoS One. 2026 Feb 10;21(2):e0315072. doi: 10.1371/journal.pone.0315072 (PMC12890121; doi:10.1371/journal.pone.0315072)
Supplement: Attachment — Submitted filename: Response to reviewers_R2.docx [file pone.0315072.s005.docx]

**Comment 1.**

I did not see any documentation provided with the repository to outline what the purpose of each file was. Addition of a README type file that provides that information I think would be helpful. That can also state the version of MATLAB used for the study and any prerequisite packages needed to run the study code.

**Response 1.**

We thank the reviewer for this helpful suggestion. As recommended, we have added a **README.txt** file to the repository. The README.txt provides a description of the purpose of each code file, specifies the MATLAB version used in the study, and lists the example data included. We believe this addition will make it easier for readers to reproduce and understand the code.

README.txt

*This repository contains the MATLAB code used in the study.*

*Note: The code was run using MATLAB R2024b.*

*[Main scripts and functions]*

*func_sto_v1.m*

*Function file implementing the tau-leaping method.*

*scp_sto_main.m*

*Main script that generates stochastic simulation runs.*

*scp_plot_check.m*

*Visualization script for the main outputs.*

*Requires pre-generated data (e.g., multishot_2_1_2.mat containing baseline simulation runs).*

*scpaux_tauleap_v1.m*

*Auxiliary script for setting up matrices and vectors used in the main code.*

*[Example data]*

*multishot_2_1_2.mat*

*Example dataset containing baseline simulation runs, used for plotting and checking outputs.*

====================================================================================

**Comment 2.**

There is a lack of comments in each code script. That makes it challenging to understand the purpose of blocks of code. A suggestion to add comments to the code scripts to help guide the user.

**Response 2.**

To address the reviewer’s concern, we have added comments to the main script (scp_sto_main.m) to clarify the structure and purpose of key code blocks. In addition, we have included a README file that describes the purpose of each script and how they are used, along with information on the MATLAB version and example data provided. We believe these additions will guide readers sufficiently in running and understanding the code, while keeping the repository streamlined.

**Comment 3.**

File ‘scp_plot_check.m’: Ran without error for me as long as I had first run the file ‘scp_sto_main.m’. However, if running the file with a clear workspace, the process errored as the prerequisite variables had not been generated. Please can amendments be made to avoid that situation (e.g. Another MAT file can be loaded that has example data to produce the plot set)?

**Response 3.**

As suggested, we have included example data (baseline scenario) as a .mat file (multishot_2_1_2.mat). This allows the reviewer to load the data directly and test the plotting script without first running the main simulation.

====================================================================================

**Comment 4.**

File ‘scpaux_tauleap_v1.m’: Also got errors attempting to run this file, even after running the main script (specifically, programme terminated on line 31 as the variable ‘scpaux_tauleap_v1’ had not been defined). Please check and provide extra information on how this file should be used by the user.

**Response 4.**

We tested the file using the repository as currently provided, and it runs without errors in our environment. However, we cannot guarantee compatibility with MATLAB versions prior to R2024b. We kindly ask the reviewer to check the MATLAB version being used and re-run the test accordingly.

====================================================================================

**Comment 5.**

For reproducibility purposes and to permit verification of model applicability, I believe more information needs to be provided on the parameterisation of social contacts, close contacts and transmission risk in the model.

I consider it reasonable that the main text reports the parameter values used. i.e. Overall basic reproduction number of six. Probability of successful disease transmission through close contact set to 60% and through social contact set to 10%. Social contacts occur at four times the frequency of close contacts.

However, I think what is then also needed is an expanded mathematical description as a section in the Supporting Information to describe the calibration of the close contact and social contact contributions to the overall reproduction number. i.e. Given “the basic reproductive number through close contact was calculated to be approximately four”, how the social contact parameters set at the stated relative contact frequency (compared to close contacts) and the successful disease transmission through social contact values results in the contribution of social contacts to the overall basic reproduction number being two.

**Response 5.**

We thank the reviewer for this helpful comment. In our study, the social contact parameters were chosen as simplifying assumptions to calibrate the overall reproduction number to approximately six, consistent with historical estimates for smallpox. Specifically, we assumed that social contacts occur at about four times the frequency of close contacts (excluding household contacts) and set the transmission probability through social contact to 10%. Together with the 60% probability for close contacts, this results in an estimated R₀ of ≈4 from close contacts and ≈2 from social contacts, yielding a total R₀ of ≈6.

To clarify this rationale, we have revised the Methods section. At the end of the relevant paragraph, we now explicitly state (L181):

*“These parameter values were chosen as simplifying assumptions to reproduce an overall basic reproductive number of approximately six, rather than as precise empirical estimates of contact-specific transmission probabilities.”*

**Comment 6.**
Helpful information has now been added into the revised manuscript to describe the sensitivity analysis. That information has all been added into the Results section. In my opinion, a substantial part of that added text is methods (L410-437). For ease of reader reference, the text describing the methodology for conducting the sensitivity analysis I would consider to be better placed at the end of the Methods section. The results subsection on the sensitivity analysis can then instead open with a high-level summary/reminder to the reader of what the purpose of the sensitivity analysis is in the context of this study.

**Response 6.**

In the revised manuscript, we have restructured the sensitivity analysis section to improve clarity. Specifically, the methodological details (L410–437) describing the use of Latin hypercube sampling, PRCC, and the parameter ranges have been moved from the Results section to the end of the Methods section under the subheading “Sensitivity Analysis”. The Results subsection on sensitivity analysis now begins with a high-level summary that emphasizes the purpose of the analysis and then presents the findings, with a reference directing the reader to the Methods section for full details.

====================================================================================

**Comment 7.**

I provided the following comment in my original review: “Recommend these are expanded to provide sufficient details so the features of each figure can be understood by the reader without having to refer to the main text. To give a couple of examples, I would suggest removing from the main text and instead placing as part of the relevant figure caption: L222-226 describing Figure 2, instead have as part of the Figure 2 caption; L322-324 describing Figure 7, instead have as part of the Figure 7 caption. Similar changes can be made to the remaining figure captions.”

The authors have actioned the couple of examples I suggested. However, I have not seen similar edits made to the other figure captions. I still view that for the other figure captions it would be helpful for more information to be given (for purpose of enabling the features of each figure to be understood by the reader without having to refer to the main text).

**Response 7.**

Following the suggestion, we have revised the figure captions to make them more self-contained, so that the main features of each figure can be understood without needing to refer to the main text:

*Figure 5. Outbreak outcomes from the baseline scenario simulations. Panels (A) and (B) show the distribution of confirmed cases by age group and in total, respectively. Panels (C) and (D) present the corresponding distributions of deaths. Results are displayed as violin plots, which illustrate both the variability and the probability density of outcomes across stochastic simulation runs.*

*Figure 6. Outbreak outcomes under different assumptions for vaccination growth rate and contact reduction, given a baseline initial vaccination number of 1,000. Results are shown on a log scale for confirmed cases (A), deaths (B), and peak severe patients (C). Each color denotes a different vaccination growth rate, while the horizontal axis indicates the level of contact reduction.*

*Figure 8. Vaccination numbers in the baseline scenario simulations. Panel (A) shows the mean daily number of vaccinations for ring vaccination (magenta) and mass vaccination (black), with the inset highlighting the early outbreak period. Panels (B) and (C) present the probability distributions for the total number of doses administered and the duration of the ring vaccination campaign, respectively, across stochastic simulation runs.*

*Figure 9. Simulation results under different vaccine prioritization strategies. Panel (A) shows daily confirmed cases, and Panel (B) shows the number of severe patients over time. The scenarios compared are: baseline (uniform vaccination, dotted blue), ascending order of age (solid orange), descending order of age (solid yellow), transmissibility-based prioritization (dashed purple), and fatality-based prioritization (dashed green).*

*Figure 10. Absolute partial rank correlation coefficients (PRCC) over time for key model parameters. Panel (A) shows PRCC values with respect to cumulative confirmed cases, and Panel (B) shows PRCC values with respect to cumulative deaths. Each color denotes a different input parameter: outbreak recognition timing (yellow), impact of social distancing (green), isolation rate for traced cases (blue), isolation rate for non-traced cases (purple), contact-identification ratio (orange), and growth rate of the daily vaccination (red). For the sensitivity analysis, parameter ranges were generated based on ±25% variability around baseline values.*

**Comment 8.**
This new figure helpfully summarises variability in outcomes under different assumptions. To enable the reader to see the underlying outcome distributions, a request to please change the box plots to be raincloud plots.

**Response 8.**

We thank the reviewer for the suggestion. In this figure, nine separate distributions are presented, and the main quantitative findings are already provided in the table and described in the text. Changing to raincloud plots would make the visualization overly complex giving disproportionate emphasis to this secondary result, which is not among the central findings of the study. In addition, since the y-axis is shown on a logarithmic scale, density shapes in raincloud plots would be visually distorted and difficult to interpret. For these reasons, we believe it is more appropriate to retain the current box plot format, which conveys the variability in a clear and concise manner.

====================================================================================

**Comment 9.**
The authors have made edits to help distinguish the different line profiles from one another. These have helped, though I think further changes would be useful to make clear to the reader the differences in outcomes between the different scenarios.

The age-based order scenarios are both solid lines. The not age-based order scenarios are both dashed lines. To help the reader distinguish between the lines in these respective pairs, a suggestion to add different marker styles to the lines (e.g. crosses, filled circles, unfilled circles, triangles)

Could present the daily case numbers and severe patient numbers on a log scale

Adding two additional panels to the figure that show the difference between each scenario compared to the baseline strategy.

- Panel C showing the differences compared to the baseline strategy for ‘Daily confirmed cases’
- Panel D showing the differences compared to the baseline strategy for ‘Severe patients’

**Response 9.**

We thank the reviewer for these detailed suggestions. At the current level of complexity, we believe the line profiles are distinguishable without additional markers, and adding markers would make the figure visually cluttered given the number of curves displayed. Presenting the outcomes on a logarithmic scale would also narrow the visual differences between scenarios, reducing the clarity of the comparison. Furthermore, the relative differences between scenarios are already summarized quantitatively in Table 5 using odds ratios. To avoid redundancy and maintain figure readability, we have chosen to retain the current figure format without additional panels.

====================================================================================

**Comment 10.**

**Figure 10 (formerly Figure 7)**
The split of this figure into two separate panels I found has made the overall figure less busy. I do have other concerns regarding accessibility, due to the line styles all being the same and some of the colours being similar. I recommend edits are made to help the reader distinguish between the different line profiles. One suggestion is to use a monochromatic colour scheme – a single colour for all the line profiles with each line profile having a different shading intensity (so that across the scenarios the line profiles range from a light shade to a dark shade).

**Response 10.**

We thank the reviewer for the suggestion. In this figure, each line represents a fundamentally different model input parameter (e.g., outbreak recognition timing, contact identification ratio, social distancing, vaccination growth rate, isolation rates). For this reason, we believe it is clearer to maintain distinct colors for each line, so that the reader can directly associate each profile with a specific intervention or epidemiological factor. Using a monochromatic color scheme with shading intensity would risk obscuring these distinctions, especially given the number of parameters included. To address accessibility, we have clarified the color-to-parameter mapping explicitly in the caption, ensuring that readers can readily distinguish the line profiles.

**Comment 11.**

L29-30: “This study develops a mathematical model to evaluate outbreak scenarios and the effectiveness of reactive intervention strategies in controlling transmission.” Please add that this evaluation is applied to the Republic of Korea.

**Response 11.**

We appreciate the reviewer’s suggestion. As recommended, we have revised the sentence in the Abstract to clarify the study setting:

*“This study develops a mathematical model to evaluate outbreak scenarios and the effectiveness of reactive intervention strategies in controlling transmission, with application to the Republic of Korea.”*

====================================================================================

**Comment 12.**

L95: “negative-pressure isolation beds to 3,500 to response to Disease-X”. Amend “response” to “respond”.

**Response 12.**

Corrected.

====================================================================================

**Comment 13.**

L244: Please add the version number of MATLAB that the study code was run with.

**Response 13.**

Corrected (2024b).

====================================================================================

**Comment 14.**
The suggested subheadings listed in the Response to Reviewers I view as sensible. However, the subheadings in the revised text appear to slightly differ. I suggest amending them to match the list below stated by the authors in the Response to Reviewers document:

- Baseline Scenario Simulation: Outbreak Outcomes
- Baseline Scenario Simulation: Impact of Outbreak Recognition on Outbreak Size
- Baseline Scenario Simulation: Vaccine Administration Over Time
- Sensitivity Analysis: Factors with Changing Influence Over Time

**Response 14.**

Corrected.

====================================================================================

**Comment 15.**

**Table 1**: A suggestion to add to the table caption that for the sensitivity analysis, a +/- 25% variability around baseline values was applied.

**Response 15.**

We thank the reviewer for the suggestion. Rather than adding this information to Table 1, we have incorporated it into the caption of Figure 10, which presents the PRCC results of the sensitivity analysis (see Response 7). We believe this placement improves clarity by linking the variability assumption directly with the figure that displays the sensitivity analysis outcomes.

**Comment 16.**

**Tables 2-4 (formerly Tables 3-5)**: As raised in my original review and by Reviewer 3, to streamline the main text these tables can be placed into Supporting Information. The reader now has Figure 6 as the main visual to analyse the results. If the reader were interested in the exact numbers, then can be signposted within the main text to look at the tables.

**Response 16.**

We appreciate the suggestion. These tables are not overly large or detailed, and one of them presents baseline results. For readers from public health backgrounds, having the numerical values directly available in the main text may be of practical interest, complementing the visual summary in Figure 6. For this reason, and to ensure accessibility for a broader readership, we have retained Tables 2–4 in the main text rather than moving them to the Supporting Information.

====================================================================================

**Comment 17.**

**Figure 4**: A comment from Reviewer 3 requested that ‘phases 1-3’ be highlighted on the figure. I did not see that detail added in the revised manuscript. I agree with Reviewer 3 that such an inclusion would provide beneficial contextual information to the reader. I therefore also request that detail is added.

**Response 17**.

have revised Figure to indicate Phases 1–3, ensuring that the three phases are clearly connected and highlighted on the figure for contextual clarity.

====================================================================================

**Comment 18.**

**Figure 5**: I thank the authors for having revised the plot style to violin plots (from box plots), which I consider to be more informative due to displaying to the reader the probability density. As suggested in my original comment, I think this plot would be further enhanced being presented as a raincloud plots*, due to a raincloud plot displaying as a combination both the probability density at different values along with jittered raw data.

*For examples of raincloud plots, see figures 3 and 4 of “Raincloud plots: a multi-platform tool for robust data visualization” [https://doi.org/10.12688/wellcomeopenres.15191.2](https://doi.org/10.12688/wellcomeopenres.15191.2?utm_source=chatgpt.com)

**Response 18.**

We appreciate the reviewer’s suggestion. While raincloud plots are indeed useful for displaying a small number of distributions, we believe they are less suitable in our case, where more than ten separate distributions are presented. Expanding this figure to incorporate raincloud plots would make the visualization very large and may unintentionally give the impression that this result represents one of the central findings of the study, whereas it is included only as supporting information. For clarity and conciseness, we therefore maintain the violin plots, which we believe provide an appropriate balance of readability and detail for this figure.
