# Supplementary material for: Smallpox outbreak scenarios and reactive intervention protocols: A mathematical model-based analysis applied to the Republic of Korea
Source: PLoS One. 2026 Feb 10;21(2):e0315072. doi: 10.1371/journal.pone.0315072 (PMC12890121; doi:10.1371/journal.pone.0315072)
Supplement: Attachment — Submitted filename: Response_to_reviewers_auresp_3.docx [file pone.0315072.s006.docx]

**Editor’s comments**

We therefore recommend the following revisions to the manuscript prior to acceptance:

- Title: Smallpox *outbreak* scenarios and reactive intervention protocols: A mathematical model-based analysis applied to the Republic of Korea

- Abstract line 1: Smallpox, caused by the variola virus, remains a potential *biosecurity* threat despite its eradication.

- Line 72: However, the presence of these viral stocks poses *biosecurity concerns, including* the potential risk of bioterrorism. Given the lack of widespread immunity in the current global population, the *accidental or* deliberate release of the smallpox virus could lead to a catastrophic outbreak.

- Line 127: Given the *potential biosecurity* threat *posed by* smallpox

- Line 137: emphasizing the need for comprehensive preparedness in the face of potential *outbreaks*.

- Line 567: The insights gained from this study provide valuable guidance to public health officials and policymakers in preparing for and responding to potential *biosecurity* threats and emerging infectious diseases

**Response to editor.**

All suggested changes have been fully incorporated into the revised manuscript.

**Reviewer #2:**

**Comment 2-1.**

Figure 10: I unfortunately do not consider the authors rebuttal to my previous comment raised on Figure 10 as sufficient – the accessibility issues on this figure I believe remain (to a lesser extent also in Figure 9).

Specifically, on the response “To address accessibility, we have clarified the color-to-parameter mapping explicitly in the caption, ensuring that readers can readily distinguish the line profiles”, if a reader has a colour-blindness condition that means they are not able to distinguish between the line colours, then the line colour information in the caption does not help in that situation (as the lines all look similar).

I therefore restate my recommendation that edits are made to this figure to help the reader distinguish between the different line profiles (given the number of lines meaning each line having a unique line style may also not be possible, that is why a monochromatic colour scheme may be a potential solution).

**Response 2-1.**

We have revised Figure 10 so that all six line profiles can now be distinguished even in grayscale or color-blind viewing conditions by using a combination of line types (solid, dashed, dotted) and distinct square markers.

**Comment 2-2.**

Comments within code scripts: Within the code files associated with the study, helpful comments have been added to the main script and a guiding README file added. Nonetheless, I disagree with the authors assessment that “We believe these additions will guide readers sufficiently in running and understanding the code” as the supporting scripts and function files lack comments. There are consequently multiple files where additional support can still be provided to the reader. Therefore, my previous comment stating “There is a lack of comments in each code script” is still to be fully resolved.

I did not see any documentation provided with the repository to outline what the purpose of each file was. Addition of a README type file that provides that information I think would be helpful. That can also state the version of MATLAB used for the study and any prerequisite packages needed to run the study code.

**Response 2-2.**

As response, we have substantially revised the README.txt. Specifically, we added header comments describing the purpose, inputs, outputs, and dependencies of every function and script. Additionally, we expanded the repository README to include a file-by-file description, execution instructions, required MATLAB version (R2024b), and necessary toolboxes. We believe these revisions provide the level of clarity requested and ensure that readers can now easily understand the structure and purpose of each script in the repository.

========README.txt=============

\# MATLAB Code Repository for the Study

This repository contains all MATLAB scripts and function files used in the stochastic simulation analyses presented in the study.

All code was executed using **MATLAB R2024b**.

\## Required MATLAB Toolboxes

The following toolboxes are required to run the simulation code:

- **Statistics and Machine Learning Toolbox**

(random number generation, probability distributions)

\## How to Run the Code

1. Run **scp_sto_main.m** to generate stochastic simulation outputs.

This script initializes parameters, sets simulation options, and calls the tau-leaping engine.

2. The main script automatically uses:

- **func_sto_v1.m** for the tau-leaping implementation

- **scpaux_tauleap_v1.m** for generating auxiliary matrices required for simulation

3. Once simulation output is generated, run **scp_plot_check.m** to visualize the results.

Example pre-generated data (`multishot_2_1_2.mat`) is included for convenience.

\## File-by-File Description

\### **1. scp_sto_main.m** — Main script (entry point)

**Purpose:**

Runs the stochastic simulation study using the tau-leaping algorithm.

**Inputs:**

- Parameter settings defined inside the script

- Auxiliary structures loaded/generated by helper functions

**Outputs:**

- `.mat` file containing the full set of stochastic simulation trajectories

- Summary variables used for visualization

**Dependencies:**

- Calls `func_sto_v1.m`

- Calls `scpaux_tauleap_v1.m`

\### **2. func_sto_v1.m** — Tau-leaping function

**Purpose:**

Implements the tau-leaping method to approximate the stochastic reaction process at each time step.

**Inputs:**

- Parameter structure

- Current state vector

- Time step size (`tau`)

- Stoichiometric matrices and rate indices from `scpaux_tauleap_v1.m`

**Outputs:**

- Updated system state after one tau step

- Reaction counts for each event type

**Dependencies:**

- Requires outputs generated by `scpaux_tauleap_v1.m`

\### **3. scpaux_tauleap_v1.m** — Auxiliary setup script

**Purpose:**

Pre-computes matrices, stoichiometric structures, rate-function indices, and bookkeeping vectors needed for tau-leaping.

**Outputs:**

- A structured set of objects used by both `func_sto_v1.m` and `scp_sto_main.m`

**Notes:**

This script is automatically called by `scp_sto_main.m` and does not need to be run independently.

\### **4. scp_plot_check.m** — Plotting and visualization

**Purpose:**

Generates plots and summary diagnostics from completed stochastic simulations.

Used for visual inspection and reproduction of key figures.

**Inputs:**

- `.mat` output generated by `scp_sto_main.m`, or

- Example dataset: `multishot_2_1_2.mat`

**Outputs:**

- Figures and visualization panels for analysis

\## Example Data

\### **multishot_2_1_2.mat**

Contains a pre-generated set of baseline stochastic simulation outputs.

Useful for testing the plotting script or reproducing examples without running full simulations.
